# Excited-State Engineering toward Accelerated Reverse Intersystem Crossing in Diindolocarbazole-Embedded Multiple-Resonance Emitters for High-Performance Blue OLEDs

**DOI:** 10.1021/acsami.5c05420

**Published:** 2025-05-16

**Authors:** Shuxin Wang, Jianping Zhou, Jibiao Jin, He Jiang, Minqiang Mai, Lian Duan, Xinping Zhang, Wai-Yeung Wong

**Affiliations:** † Institute of Information Photonics Technology, School of Physics and Optoelectronic Engineering, 12496 Beijing University of Technology, Beijing 100124, P. R. China; ‡ Department of Applied Biology and Chemical Technology and Research Institute for Smart Energy, 26680 The Hong Kong Polytechnic University, Hung Hom, Hong Kong 999077, P. R. China; § The Hong Kong Polytechnic University Shenzhen Research Institute, Shenzhen 518057, P. R. China; ∥ Key Lab of Organic Optoelectronics and Molecular Engineering of Ministry of Education, Department of Chemistry, 12442 Tsinghua University, Beijing 100084, P. R. China

**Keywords:** blue organic light-emitting diodes, diindolocarbazole-embedded multiple-resonance emitters, excited-state engineering, reverse intersystem crossing, thermally activated delayed fluorescence

## Abstract

Simultaneously achieving a narrow emission band, high efficiency, and excellent color purity remains a formidable challenge in the development of blue organic light-emitting diodes (OLEDs). Diindolocarbazole-embedded multiple-resonance emitters show great potential owing to their extremely narrow emission band, but the practical applications are severely limited by the slow reverse intersystem crossing rate (*k*_RISC_) with the order of magnitude value of 10^2^. Herein, we present an effective strategy to accelerate the RISC process by acceptor decoration to regulate the excited state. Through modulating the electron-withdrawing ability from TFB to TPT, the long-range charge transfer excited state is successfully induced, which leads to the decreased Δ*E*_ST_ and increased spin–orbital coupling (SOC) matrix elements, contributing to the dramatically accelerated *k*_RISC_ up to 1.11 × 10^4^ s^–1^ for pICz-TPT. Moreover, the narrowband blue emission is basically retained for the proof-of-concept pICz-TPT with an emission peak at 449 nm, a full width at half-maximum of 44 nm, and CIE coordinates of (0.15, 0.10). Impressively, the nonsensitized OLEDs based on the pICz-TPT emitter exhibit the highest maximum external quantum efficiency (EQE_max_) of 14.4% among all the reported blue OLEDs on the basis of pICz derivatives (which typically remained below 5%), and a further boost of efficiency with EQE_max_ of 24.2% is realized in the hyperfluorescent OLEDs. This work provides a powerful design tool toward highly efficient emitters with good color purity.

## Graphical abstract

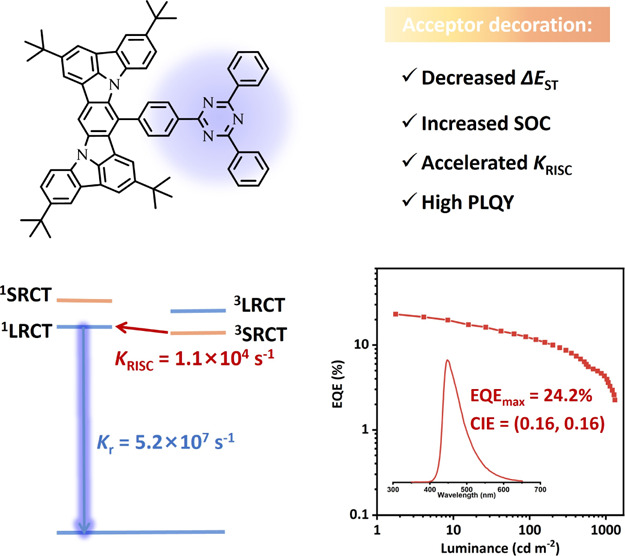

## 1 Introduction

Developing highly efficient blue organic light-emitting diodes (OLEDs) with superior color purity remains an ongoing pursuit for wide-gamut and high-resolution displays.^1–4^ Thermally activated delayed fluorescence (TADF) emitters have sparked tremendous interest since they can utilize the reverse intersystem crossing (RISC) process to harness both singlet and triplet excitons, enabling a theoretical 100% internal quantum efficiency.^5–7^ To facilitate the efficient RISC, conventional TADF emitters generally incorporate donor–acceptor (D–A) architectures to diminish the energy gap (Δ*E*_ST_) between the lowest excited singlet (S_1_) and triplet (T_1_) states through minimizing the spatial overlap of the highest occupied (HOMO) and lowest unoccupied (LUMO) molecular orbitals.^8–10^ However, the twisted conformation of D–A molecules inevitably causes remarkable structural relaxation in the S_1_ state and significant vibronic coupling between the ground state (S_0_) and S_1_, resulting in a broad emission profile with the full width at half-maximum (fwhm) typically exceeding 70 nm and thereby poor color purity.^11,12^

Currently, remarkable advancements in multiple-resonance (MR) organic emitters have unlocked exciting opportunities for the development of next-generation blue OLEDs.^13–18^ By integrating opposite resonating features of the electron-withdrawing and electron-donating units within a rigid polycyclic scaffold, the alternative distributions of the frontier molecular orbitals (FMOs) can be triggered by the MR effect, which effectively suppresses the structural relaxation and the vibronic coupling between S_0_ and S_1_, resulting in a narrowband emission and guaranteed color purity.^19–21^ However, MR emitters generally suffer from an inefficient RISC process with the RISC rate (*k*_RISC_) on the order of magnitude value of 10^4^ s^–1^ in contrast to conventional TADF emitters with *k*_RISC_ on the order of magnitude value of 10^5^ s^–1^ to 10^7^ s^–1^.^22–25^ The TADF sensitizer assistant is regarded as an effective strategy to harvest the triplet excitons for high-performance OLEDs, and a high maximum external quantum efficiency (EQE_max_) above 30% has been achieved.^26–28^ However, the TADF sensitizer cooperation inevitably broadens the emission band and causes energy loss of the devices.^29^ It is also proved that embedding heavy elements into the MR skeleton can promote the spin–orbit coupling (SOC) and thus accelerate *k*_RISC_.^30–33^ Nonetheless, the cooperation of the heavy elements generally broadens the emission spectrum since their large atomic radius inevitably leads to structural variations between ground and excited states. Inspiringly, several recent studies propose that *k*_RISC_ of MR emitters can be accelerated without the sacrifice of color purity by mixing wave functions with different natures, and the *k*_RISC_ of B,N-MR emitters has been promoted to be above 10^6^ s^–1^.^34,35^

The diindolocarbazole (pICz)-based MR emitters show great potential for practical applications due to their advantages of deep blue emission with an especially narrow emission band (fwhm ≤ 14 nm) and pure color purity (CIE_*y*_ ≤ 0.05) and a mild synthesis procedure with high yields.^26,36–38^ However, pICz and its derivatives suffer from particularly large Δ*E*_ST_ (above 0.29 eV) and insufficient triplet exciton utilization (with *k*_RISC_ on the order of 10^2^ s^–1^), as summarized in Table S1. By virtue of the tetramesitylating decoration, Hall et al. reduced Δ*E*_ST_ to 0.26 eV for the DiICzMes4 emitter, but the low delayed emission ratio (1.2%) leads to the slow *k*_RISC_ of 1.8 × 10^2^ s^–1^.^37^ Through incorporating the electron donor in pICz to induce resonant spin-vibronic coupling, Lee et al. reduced Δ*E*_ST_ to 0.27 eV and enhanced *k*_RISC_ to 1.4 × 10^3^ s^–1^.^38^ However, the *k*_RISC_ of pICz derivatives remains insufficient to effectively utilize the triplet excitons due to their inherently large Δ*E*_ST_. Therefore, more explorations on effective strategies toward efficient RISC in pICz-based MR emitters are imperative for achieving pure blue OLEDs with extraordinary performance.

Herein, a novel strategy to enhance the *k*_RISC_ of pICz-based MR emitters was developed. By integrating the merits of D–A TADF emitters and MR emitters, we proposed that peripheral decoration of electron acceptors on the pICz core could regulate the excited states, giving rise to the controlled Δ*E*_ST_ toward accelerated *k*_RISC_. To validate our design strategy, we successfully synthesized two narrowband blue emitters pICz-TFB and pICz-TPT by incorporating (trifluoromethyl)­benzene (TFB) and 2,4,6-triphenyl-1,3,5-triazine (TPT) with different electron-withdrawing abilities into the pICz core. By virtue of theoretical calculations, it was demonstrated that the long-range charge transfer (LRCT) excited state could be induced through the cooperation of TPT with strong electron-withdrawing ability, giving rise to the decreased Δ*E*_ST_ and increased SOC matrix elements. As expected, pICz-TPT shows decreased Δ*E*_ST_ and accelerated *k*_RISC_ in contrast to pICz-TFB. Consequently, the nonsensitized OLEDs based on the pICz-TPT emitter exhibit the highest EQE_max_ of 14.4% among all the reported blue OLEDs on the basis of pICz derivatives (which typically remained below 5%). Moreover, the device optimization was executed with the TADF sensitizer assistant, and a high EQE_max_ of 24.2% and good color purity with CIE coordinates of (0.16, 0.16) were achieved for pure blue OLEDs with a pICz-TPT emitter. This work highlights the critical role of excited-state engineering in accelerating *k*_RISC_, providing inspiration for the rational design of highly efficient MR emitters for pure blue OLEDs with outstanding performance.

## 2 Experimental Section

### 2.1 Synthesis of pICz-TFB

((Trifluoromethyl)­phenyl)­boronic acid (1.9 g, 10 mmol), pICz-Br (1.6 g, 2 mmol), Pd­(PPh_3_)_4_ (0.23 g, 0.2 mmol), K_2_CO_3_ (2 M, 10 mL), and ethanol (10 mL) were dissolved in toluene (150 mL). The reaction mixture was heated to reflux for 12 h under a nitrogen atmosphere. After the reaction was completed, the solvents were evaporated under vacuum, and the residue was extracted using dichloromethane (DCM) followed by washing with deionized water. The organic layer was dried using Na_2_SO_4_ and evaporated to complete dryness. The pICz-TFB was attained as a yellow powder through silica gel chromatography purification (1.13 g, 73% yield). ^1^H NMR for pICz-TFB (400 MHz, chloroform-*d*): δ (ppm) 8.62 (s, 1H), 8.33 (d, *J* = 1.2 Hz, 1H), 8.20 (d, *J* = 1.4 Hz, 2H), 8.12–8.07 (m, 4H), 8.05–8.00 (m, 3H), 7.67 (dd, *J* = 8.5, 1.9 Hz, 1H), 7.08 (dd, *J* = 8.8, 2.1 Hz, 1H), 6.86 (d, *J* = 1.2 Hz, 1H), 5.54 (d, *J* = 8.7 Hz, 1H), 1.64 (s, 9H), 1.52 (s, 9H), 1.40 (s, 9H), 1.35 (s, 9H). ^13^C NMR for pICz-TFB (101 MHz, chloroform-*d*): δ 146.69, 146.34, 144.69, 144.46, 143.97, 137.80, 137.17, 134.53, 134.27, 131.90, 131.52, 130.01, 129.98, 129.89, 125.94, 125.90, 124.10, 123.74, 121.68, 119.77, 119.12, 118.41, 118.24, 117.81, 117.73, 117.27, 116.91, 116.54, 115.89, 112.83, 111.53, 105.33, 36.00, 35.65, 34.94, 34.65, 32.86, 32.45, 31.95, 31.77. MALDI-TOF *m*/*z*: calcd for C_53_H_51_F_3_N_2_ [M]^+^, 772.400; found, 772.540.

### 2.2 Synthesis of pICz-TPT

pICz-TPT was synthesized via the same procedure as pICz-TFB using 2,4-diphenyl-6-(4-(4,4,5,5-tetramethyl-1,3,2-dioxaborolan-2-yl)­phenyl)-1,3,5-triazine and pICz-Br as starting materials. The pICz-TPT was attained as a yellow powder with a yield of 78%. ^1^H NMR for pICz-TPT (400 MHz, chloroform-*d*): δ (ppm) 9.28–9.22 (m, 2H), 8.93–8.88 (m, 4H), 8.64 (s, 1H), 8.35 (d, *J* = 1.2 Hz, 1H), 8.21 (t, *J* = 1.7 Hz, 2H), 8.14–8.10 (m, 3H), 8.10–8.03 (m, 2H), 7.71–7.60 (m, 7H), 7.14 (d, *J* = 1.2 Hz, 1H), 7.06 (dd, *J* = 8.8, 2.1 Hz, 1H), 5.83 (d, *J* = 8.7 Hz, 1H), 1.66 (s, 9H), 1.53 (s, 9H), 1.34 (s, 9H), 1.33 (s, 9H). ^13^C NMR for pICz-TPT (101 MHz, chloroform-*d*): δ 171.97, 171.40, 146.57, 146.33, 144.58, 144.56, 144.27, 144.01, 143.14, 138.03, 137.20, 136.78, 136.32, 134.69, 134.54, 132.70, 131.74, 130.02, 129.94, 129.54, 129.13, 128.79, 128.65, 124.04, 124.02, 122.87, 119.73, 118.94, 118.50, 118.14, 118.08, 117.92, 117.79, 116.73, 116.43, 115.80, 113.34, 111.51, 105.13, 36.02, 35.70, 34.95, 34.65, 32.90, 32.59, 31.98, 31.77. MALDI-TOF *m*/*z*: calcd for C_53_H_51_F_3_N_2_ [M]^+^, 935.493; found, 935.354.

## 3 Results and Discussion

### 3.1 Molecular Design and Simulation

MR emitters with short-range charge transfer (SRCT) generally exhibit superior color purity but inferior *k*_RISC_ in contrast to conventional D–A TADF emitters with LRCT.^34^ Herein, we incorporate an electron acceptor into the rigid pICz core to integrate the high color purity of the MR emitter and the efficient RISC of the D–A configuration. As depicted in Figure 1, TFB and TPT with different electron-withdrawing abilities are selected to modulate the CT transition character toward accelerated *k*_RISC_. Moreover, the asymmetric structure is constructed with only one peripheral unit to improve the solubility of the rigid pICz derivatives.

**Figure 1:**
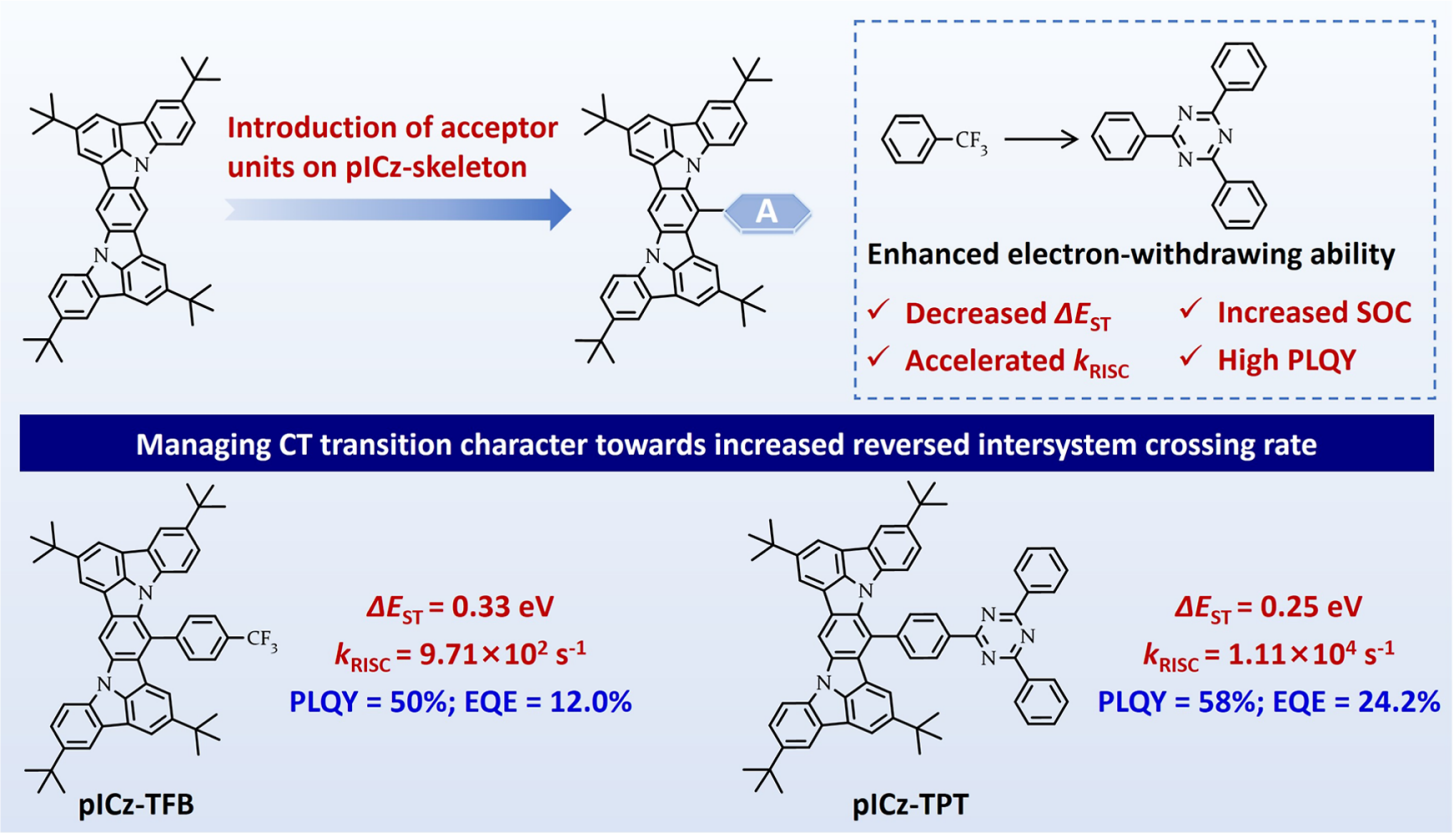
Design strategy for pICz-TFB and pICz-TPT toward accelerated *k*_RISC_.

To evaluate the impact of the acceptor decoration on the optoelectronic properties of the pICz derivatives, density functional theory (DFT) and time-dependent DFT (TD-DFT) calculations at the b3lyp/6–31g­(d) level were performed utilizing the Gaussian program. Figure 2a illustrates the calculated FMO distributions and energy-level diagrams for pICz-TFB and pICz-TPT. Surprisingly, two molecules exhibit distinct FMO distributions. For pICz-TFB, the FMOs are predominantly localized on the pICz skeleton with alternate atomic distribution patterns, demonstrating a typical MR-type configuration. In sharp contrast, pICz-TPT shows FMO distributions with the feature of conventional D–A TADF emitters with the HOMO and LUMO predominantly localized on the pICz core and peripheral TPT acceptor, respectively. The distinct separation of HOMO and LUMO distributions results from the stronger electron-withdrawing ability of TPT. The further analysis of natural transition orbital (NTO) distributions demonstrates that the SRCT and LRCT excitations dominate the S_1_ state of pICz-TFB and pICz-TPT, respectively, validating that acceptor modulation is an effective strategy to engineer the excited-state character (Figure S1). Differently, two emitters show similar NTO distributions for the T_1_ state with alternate atomic distributions on the pICz skeleton (Figure S1). Consequently, the S_1_ energy level of pICz-TPT is significantly lower than that of pICz-TFB, while the T_1_ energy level for the two emitters is similar, which contributes to the greatly reduced Δ*E*_ST_ (0.01 eV) of pICz-TPT compared with pICz-TFB (0.38 eV). The SOC matrix elements were also calculated since strong SOC is also crucial for efficient RISC apart from the small Δ*E*_ST_ according to Fermi’s golden rule. As shown in Figure 2b, the SOC matrix element between the S_1_ and T_1_ of pICz-TPT (⟨S_1_|Ĥ_SOC_|T_1_⟩ = 0.73) is dramatically larger than that for pICz-TFB (⟨S_1_|Ĥ_SOC_|T_1_⟩ = 0.01), which can be attributed to the significant discrepancy in the excitation characters between the S_1_ and T_1_ states of pICz-TPT.^34,35^ The significantly enhanced SOC strength, together with decreased Δ*E*_ST_, indicates the potential of pICz-TPT for increased *k*_RISC_.

**Figure 2:**
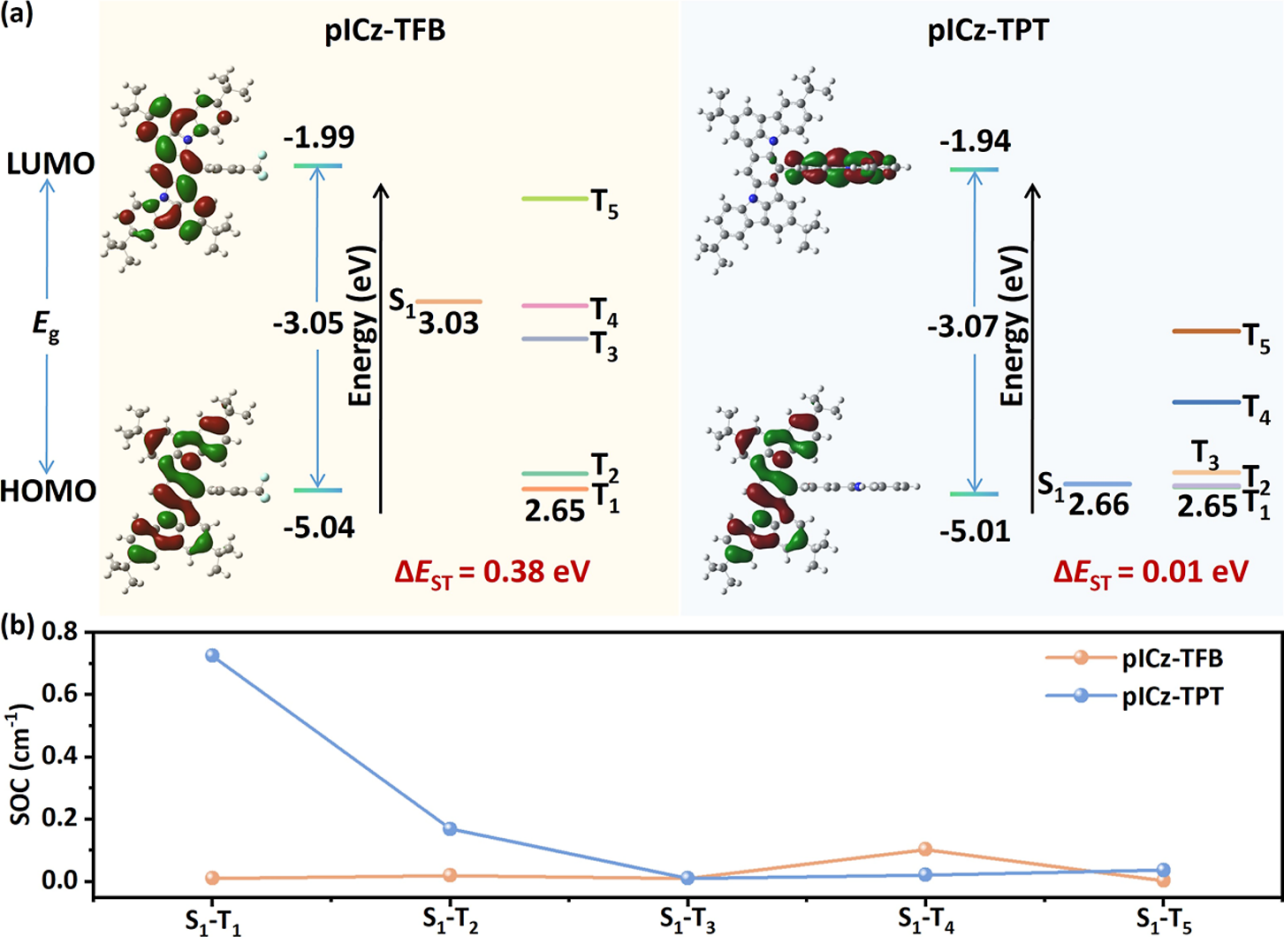
(a) FMO distributions and energy-level diagram and (b) SOC matrix elements between S_1_ and T_n_ states of pICz-TFB and pICz-TPT.

### 3.2 Synthesis and Characterization

The synthesis procedure avoids the use of reactive alkyllithium reagents and achieves high yields, as shown in Scheme S1. The pICz-Br was prepared utilizing the previously reported method,^26^ followed by the reaction with (4-(trifluoromethyl)­phenyl)­boronic acid and 2,4-diphenyl-6-(4-(4,4,5,5-tetramethyl-1,3,2-dioxaborolan-2-yl)­phenyl)-1,3,5-triazine to yield the desired products pICz-TFB and pICz-TPT, respectively. Notably, the dehalogenation of the aryl halide occurs during the Suzuki–Miyaura reaction, leading to the monoaryl-substituted final compounds. After oxidative addition, the Pd complex oxidizes ethanol to gain a hydride ligand. The aryl and hydride subsequently undergo reductive elimination, yielding the dehalogenation product.^39^ The ^1^H/^13^C NMR spectroscopy and mass spectrometry were conducted to characterize the molecular structures, and the detailed data were elaborated in the Experimental Section and Supporting Information (Figures S2–S8). Owing to the asymmetric structure configuration and the large steric hindrance introduced by the TFB and TPT units, pICz-TFB and pICz-TPT manifest good solubility in toluene, ethyl acetate, dichloromethane, etc.

### 3.3 Thermal and Electrochemical Properties

The thermogravimetric analysis (TGA) measurements were performed to investigate the thermal properties. As displayed in Table 1 and Figure S9, pICz-TFB and pICz-TPT demonstrate exceptional thermal stability. The decomposition temperature (*T*_d_, defined as 5% weight loss) is up to 473 and 542 °C for pICz-TFB and pICz-TPT, respectively, which is beneficial for their practical applications in OLEDs. The electrochemical properties were investigated by electrochemical cyclic voltammetry (CV) measurements (Figure S10). The HOMO energy levels of pICz-TFB and pICz-TPT are estimated to be −5.35 eV and −5.32 eV, respectively, based on the onset oxidation potential of the CV curves (Equation S1, Table 1). Correspondingly, the LUMO energy levels are determined as −2.54 eV and −2.53 eV, derived from HOMO – *E*_g_, where *E*_g_ represents the bandgap energy estimated from the absorption spectra onset (2.81 eV for pICz-TFB, 2.79 eV for pICz-TPT, Figure 3a).

**Table 1:** Photophysical, Electrochemical, and Thermal Properties of pICz-TFB and pICz-TPT

compound	λ_abs_ ^a^ (nm)	λ_em_ ^a^ (nm)	fwhm^a^ (nm)	PLQY^b^ (%)	τ_p_ ^b^ (ns)	τ_d_ ^b^ (ms)	CIE^a^ (*x*, *y*)	*E*_g_^a^^,^^c^ (eV)	*E*_S1_^a^^,^^d^ (eV)	*E*_T1_^a^^,^^d^ (eV)	HOMO/LUMO^e^ (eV)	*T*_d_^f^ (°C)
pICz-TFB	363, 430	438	18	50	6.72	1.43	(0.16, 0.03)	2.81	2.81	2.48	–5.35/–2.54	473
pICz-TPT	363, 430	449	44	58	4.08	0.25	(0.15, 0.10)	2.79	2.73	2.48	–5.32/–2.53	542

a Measured in 10^–5^ mol L^–1^ toluene.

b Photoluminescence quantum yield (PLQY), prompt lifetime (τ_p_), and delayed lifetime (τ_d_) measured in doped films with 1% emitter in the PPF host.

c Optical energy gap derived from the onset of the absorption spectra.

d The energy level of the lowest excited singlet (*E*_S1_) and triplet states (*E*_T1_) derived from the fluorescence and phosphorescent spectra, respectively.

e HOMO derived from the onset oxidation potential of the CV curves; LUMO derived from HOMO – *E*_g_.

f Decomposition temperature determined from TGA measurements.

**Figure 3:**
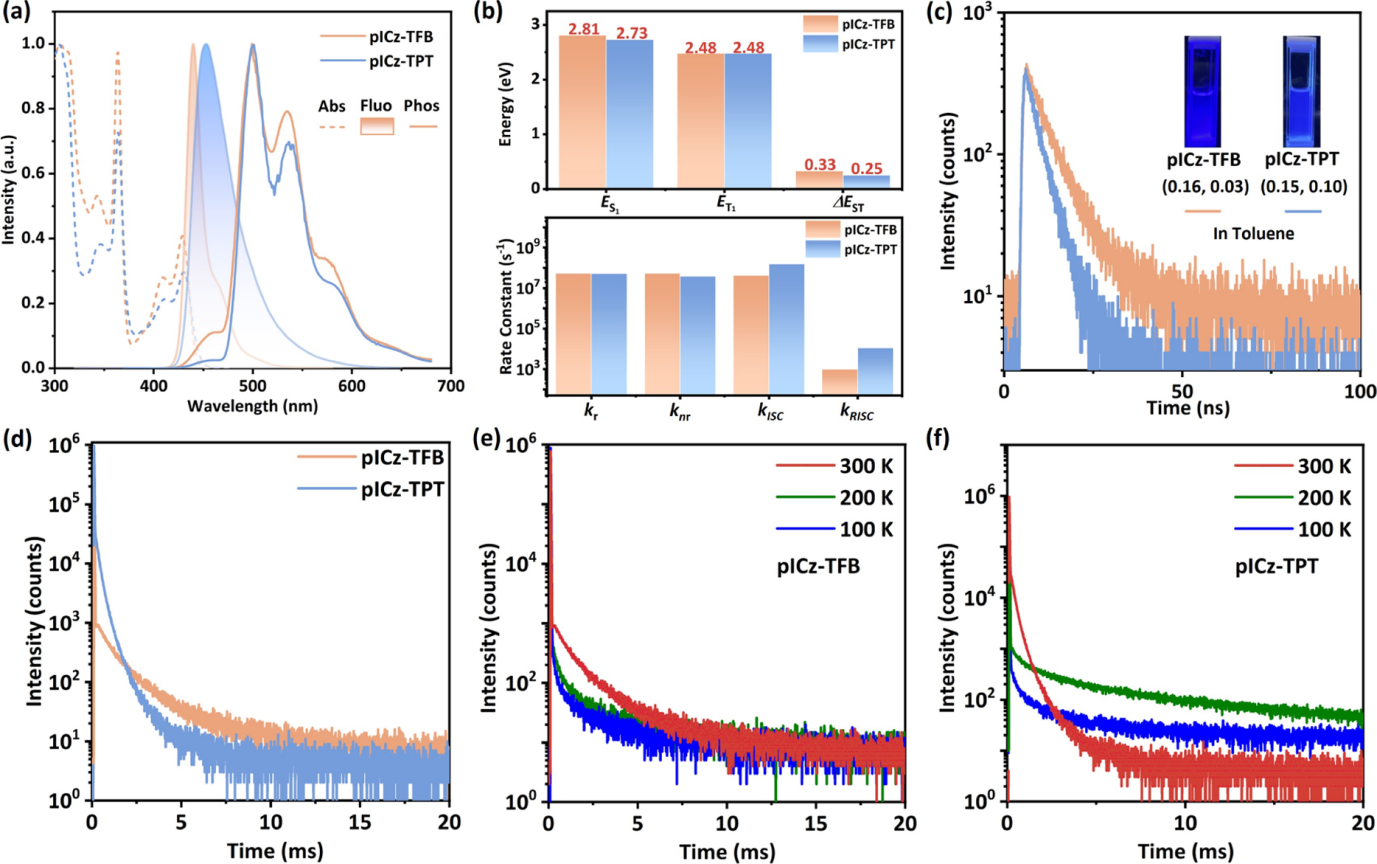
(a) The UV–vis absorption, fluorescence, and phosphorescent spectra (77 K) of pICz-TFB and pICz-TPT (10^–5^ mol L^–1^ in toluene). (b) The energy levels and the key rate constants of pICz-TFB and pICz-TPT. (c) Prompt, (d) delayed, and (e,f) temperature-dependent photoluminescence decay curves of PPF:1% pICz-TFB- and PPF:1% pICz-TPT-doped films.

### 3.4 Photophysical Properties

The photophysical properties of pICz-TFB and pICz-TPT were comprehensively investigated in 1 × 10^–5^ mol L^–1^ toluene. As illustrated in Figure 3a, two emitters show similar absorption properties, consistent with that of the prior reported pICz-based emitters.^26,29,37,38^ The absorption bands in the low-energy region (370–450 nm) are assigned to the n–π* transitions of the ICz component, while those in the high-energy region (below 370 nm) are associated with the π–π* transitions of the fused molecular architecture.^40,41^ Owing to their rigid structure, pICz-TFB and pICz-TPT present narrowband blue emission with peak wavelengths of 438 and 449 nm and CIE coordinates of (0.16, 0.03) and (0.15, 0.10), respectively. The pICz-TFB exhibits a shoulder band at the lower energy (∼475 nm), a feature that has also been observed in some reported MR molecules.^15,42^ Based on Franck–Condon analysis, it results from the structural variations between the S_0_ and S_1_ states, corresponding to the 0–1 vibronic transition from vibrational excited state *v*_1_ = 0 in S_1_ to vibrational ground state *v*_0_ = 1 in S_0_.^43,44^ Notably, the difference in the excited-state configurations brings about profound consequences for their emission properties. Compared to pICz-TFB with a Stokes shift of 9 nm and fwhm of 18 nm, pICz-TPT shows a larger Stokes shift of 19 nm and fwhm of 44 nm, which can be ascribed to its LRCT excitation character. Upon the solvent modulation from low-polarity toluene to high-polarity dichloromethane (Figure S11), pICz-TFB exhibits a negligible maximum emissive bathochromic shift, while that of pICz-TPT is as large as 77 nm, further attesting to the dominant role of SRCT transition and LRCT transition in the S_1_ state of pICz-TFB and pICz-TPT, respectively. By comparing the differences between their fluorescence and phosphorescence spectral peak maximum, the Δ*E*_ST_ for pICz-TPT is estimated to be 0.25 eV (Figure 3b), which is not only smaller than that of pICz-TFB (0.33 eV) but also the smallest among all the reported pICz derivatives, as summarized in Table S1. The obviously reduced Δ*E*_ST_ of pICz-TPT can be credited to the lowered S_1_ state induced by the TPT with a strong electron-withdrawing capability.

To explore the intersystem crossing process, we further gain insights into the photophysical properties of PPF:1% pICz-TFB- and PPF:1% pICz-TPT-doped films, where PPF denotes dibenzo­[*b*,*d*]­furan-2,8-diylbis­(diphenylphosphine oxide). Their fluorescence spectra show a slightly red-shifted emission band and broadened fwhm compared with those of their toluene solution (Figure S12), which can be ascribed to the aggregation effect of the dopant and the solid solvation effect of the host.^45^ pICz-TFB- and pICz-TPT-doped films exhibit photoluminescence quantum yields (PLQYs) of 50% and 58%, respectively. The transient decay curves of the doped films are displayed in Figure 3c,d. Two emitters show rapid, prompt decay accompanied by a delayed component, which indicates their TADF character. To further verify this, we also measured the transient decay curves across various temperatures. As the temperature rises, the delayed component is gradually intensified (Figure 3e,f), further confirming their TADF character. Based on the above experimental results, the key kinetic constants are extrapolated and summarized in Figure 3b and Table S2 (Equations S2–S8). Two emitters show a fast radiative decay rate (*k*_r_) of 5.4 × 10^7^ s^–1^ and 5.2 × 10^7^ s^–1^ for pICz-TFB and pICz-TPT, respectively, which is comparable to that of the reported MR-TADF emitters.^46^ In particular, pICz-TPT exhibits a higher delayed ratio of 64% and a shorter delayed lifetime of 0.25 ms compared to pICz-TFB (delayed ratio of 28%, delayed lifetime of 1.43 ms), contributing to the significantly accelerated *k*_RISC_ of 1.1 × 10^4^ s^–1^ for pICz-TPT compared to that of 9.7 × 10^2^ s^–1^ for pICz-TFB. The significantly accelerated *k*_RISC_ of pICz-TPT can be ascribed to its reduced Δ*E*_ST_ and increased SOC matrix elements introduced by LRCT character, demonstrating the positive effect of excited-state engineering on accelerating *k*_RISC_. It is worth mentioning that both emitters show higher *k*_RISC_ than the original pICz with that of 1.5 × 10^2^ s^–1^,^38^ and the *k*_RISC_ of pICz-TPT is the highest among all the reported blue pICz derivatives (Table S1), which verifies that acceptor modulation is a reliable approach to engineer the excited state toward an efficient RISC process.

### 3.5 Electroluminescence Properties

To evaluate the electroluminescence (EL) properties of two emitters, OLEDs were fabricated with the device configuration of ITO (indium tin oxide)/TAPC (1,1-bis­[4-[*N*,*N*′-di­(*p*-tolyl)­amino]­phenyl]­cyclohexane, 30 nm)/TCTA (4′,4′-tris­(carbazol-9-yl)-triphenylamine, 5 nm)/mCP (1,3-di-9-carbazolylbenzene, 5 nm)/EMLs (20 nm)/PPF (5 nm)/Bphen (4,7-diphenyl-1,10-phenanthroline, 30 nm)/LiF (0.5 nm)/Al (150 nm). PPF is adopted as the host matrix for the emitting layer because of its well-matched energy levels and excellent charge-transporting mobility.^47^ Figure 4a–d illustrates the EL performance of the fabricated OLEDs, with key parameters summarized in Table 2. The OLEDs utilizing pICz-TPB and pICz-TPT as emitters showcase narrowband blue emission with peak wavelengths at 441 and 445 nm, fwhm of 21 and 48 nm, and corresponding CIE coordinates of (0.15, 0.04) and (0.17, 0.16), respectively. Although the fwhm of EL spectrum for pICz-TPT is relatively broader in contrast to that of pICz-TPB due to its LRCT character, it is still narrower than that of conventional D–A TADF emitters (typically exceeding 70 nm)^11,12^ and comparable to the prior reported OLEDs based on pICz and pICz-TTA emitters with fwhm of 41 and 30 nm, respectively.^26^ Strikingly, without TADF sensitizer assistance, pICz-TPT OLEDs realize a remarkably high EQE_max_ of 14.4%, which not only far exceeds pICz-TPB OLEDs with EQE_max_ of 2.2% in this work but also significantly surpasses the prior reported OLEDs based on pICz derivatives with EQE_max_ below 5% (Table S1).^29,37^ The high efficiency of pICz-TPT OLEDs can be attributed to the accelerated *k*_RISC_ and high PLQY introduced by the LRCT character, demonstrating that excited-state engineering could serve as an effective strategy to accelerate *k*_RISC_ toward high-performance OLEDs.

**Figure 4:**
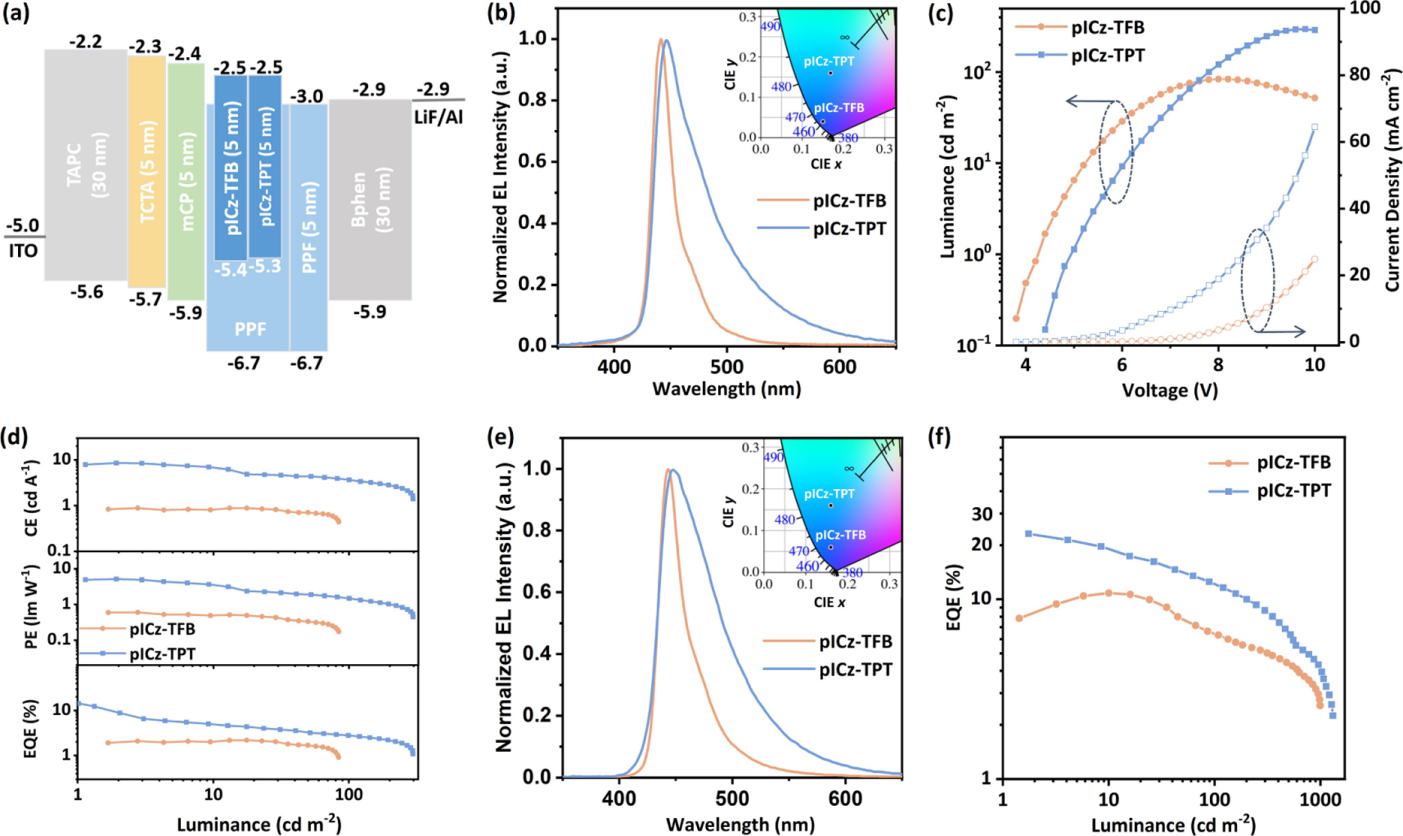
(a) Device configuration, (b) electroluminescence performance, (c) luminance and current density versus voltage, and (d) efficiency versus luminance plots of the nonsensitized OLEDs with PPF:1 wt % pICz-TPB/pICz-TPT as the emitting layer. (e) Electroluminescence performance and (f) external quantum efficiency versus luminance plots of the hyperfluorescent OLEDs with the emitting layer of PPF:30 wt % m4TCzPhBN:1 wt % pICz-TPB/pICz-TPT.

**Table 2:** Electroluminescence Performances of pICz-TPB and pICz-TPT

device	emitter	λ_EL_ ^a^ (nm)	fwhm (nm)	EQE^b^ (%)			CIE (*x*,*y*)	*V*_on_^c^ (V)	PE_max_ ^d^ (lm·W^–1^)	CE_max_ ^e^ (cd·A^–1^)
				max	100 cd m^–2^	1000 cd m^–2^				
OLEDs	pICz-TFB	441	21	2.2	-	-	(0.15, 0.04)	4.2	0.6	0.8
	pICz-TPT	445	48	14.4	2.9	-	(0.17, 0.16)	4.8	5.2	8.5
HF OLEDs	pICz-TFB	443	26	12.0	6.4	2.5	(0.15, 0.06)	3.6	5.7	7.0
	pICz-TPT	447	58	24.2	12.3	4.3	(0.16, 0.16)	3.4	25.4	27.5

a EL emission peak.

b External quantum efficiency at different luminance.

c Turn-on voltage at 1 cd m^–2^.

d Maximum power efficiency.

e Maximum current efficiency.

Even though the *k*_RISC_ of pICz-TPT has been considerably accelerated, it is still inferior, in contrast to conventional D–A TADF emitters. To further optimize the OLED performance, the hyperfluorescent (HF) OLEDs were fabricated to efficiently utilize the triplet excitons. 2′,4′,5′,6′-Tetrakis­(3,6-di-*tert*butyl-9H-carbazol-9-yl)-[1,1′:3′,1″-terphenyl]-4,4″-dicarbonitrile (m4TCzPhBN) is selected as the TADF sensitizer due to its high *k*_RISC_ and appropriate energy levels.^48^ As shown in Figure S13, after charge recombination on the TADF sensitizer, the triplet excitons undergo upconversion to singlet states through the RISC process. These singlet excitons subsequently transfer their energy to the emitter via Förster resonance energy transfer (FRET), facilitating efficient exciton utilization in the devices.^26,49^ The complete energy transfer from the sensitizer to the emitters for the PPF:30% m4TCzPhBN:1% pICz-TFB/pICz-TPT system can be verified by their obviously shortened delayed lifetime in the order of microseconds and identical emission spectra with PPF:1% pICz-TFB/pICz-TPT (Figures S14 and S15).

As expected, the performance of the OLED has been significantly boosted, as shown in Figures 4e,f and S16. The HF OLEDs with pICz-TPT and pICz-TFB present high EQE_max_ values of 24.2% and 12.0%, respectively. The pICz-TFB HF OLEDs remain a narrowband emission character with a peak wavelength of 443 nm, fwhm of 26 nm, and CIE coordinates of (0.15, 0.06). Although the emission band of pICz-TPT HF OLEDs is relatively broader with a fwhm of 58 nm, the peak wavelength of 447 nm leads to the CIE coordinates of (0.16, 0.16), demonstrating the good color purity for blue emission. The HF OLEDs show a relieved efficiency roll-off, as shown in Table S3, which can be attributed to the fast RISC process facilitated by the TADF sensitizer. Although pICz-TPT shows higher *k*_RISC_ values than pICz-TFB, the efficiency roll-off behavior of their HF OLEDs is similar, with ∼50% roll-off at 100 cd m^–2^ and ∼80% roll-off at 1000 cd m^–2^. The similarity occurs because the triplet exciton harvesting in the emitting layer of HF OLEDs is dominated by the TADF sensitizer due to its significantly higher *k*_RISC_ value of 10^6^ s^–1^. The pICz-TFB and pICz-TPT mainly functionalize as the terminal fluorescence emitter only.^50^ Consequently, the impact of *k*_RISC_ of the pICz-TFB and pICz-TPT on the efficiency roll-off behavior of HF OLEDs is negligible. It is worth noting that the HF OLEDs with pICz-TFB and pICz-TPT emitters show better efficiency roll-off behavior than the pICz HF OLEDs, which could be ascribed to their more balanced charge carrier mobility contributed by the peripheral TFB and TPT units with good electron-transporting ability.^29^

## 4 Conclusion

In summary, we have presented an effective molecular design strategy to accelerate the RISC process in pICz-based MR emitters by incorporating an appropriate electron acceptor to regulate the excited state. Through modification of the pICz core with TFB and TPT, two emitters pICz-TFB and pICz-TPT have been successfully developed to explore the impact of the electron-withdrawing ability on their overall properties. Benefiting from the rigid structure, pICz-TFB and pICz-TPT exhibit narrowband blue emission with peak wavelengths of 438 and 449 nm, fwhm of 18 and 44 nm, and CIE coordinates of (0.16, 0.03) and (0.15, 0.10), respectively. Theoretical calculations have revealed that the strong electron-withdrawing ability of TPT could induce the LRCT excited state, endowing pICz-TPT with the decreased Δ*E*_ST_ and increased SOC matrix elements. Detailed analysis of the photophysical properties further demonstrated the decreased Δ*E*_ST_ and accelerated *k*_RISC_ of pICz-TPT in contrast to pICz-TFB and prototypical pICz. Consequently, the nonsensitized OLEDs based on the pICz-TPT emitter exhibit the highest EQE_max_ of 14.4% among all the reported blue OLEDs on the basis of pICz derivatives (which typically remained below 5%). Moreover, with the assistance of a TADF sensitizer, a high EQE_max_ of 24.2% and CIE coordinates of (0.16, 0.16) have been achieved for pure blue OLEDs based on pICz-TPT. Our work attests to the fact that acceptor modulation is an effective strategy to engineer the excited state toward accelerated *k*_RISC_, promoting the design of MR emitters with high efficiency and good color purity.

## Supplementary Material



## Notes

Additional experimental details, including measurement methods, theoretical calculation results, photophysical properties, device fabrication, and nuclear magnetic resonance spectra (PDF)
