# Supplementary material for: Excited-State Engineering toward Accelerated Reverse Intersystem Crossing in Diindolocarbazole-Embedded Multiple-Resonance Emitters for High-Performance Blue OLEDs
Source: ACS Appl Mater Interfaces. 2025 May 16;17(21):31192–200. doi: 10.1021/acsami.5c05420 (PMC12123562; doi:10.1021/acsami.5c05420)
Supplement: Supplementary file 1 [file am5c05420_si_001.pdf]

Supporting Information

Excited state engineering toward accelerated reverse intersystem crossing in diindolocarbazole-embedded multiple-resonance emitters for high-performance blue OLEDs

Shuxin Wang,[ab] Jianping Zhou,[c] Jibiao Jin,[b] He Jiang,[b] Minqiang Mai,[c] Lian Duan,[c] Xinping Zhang[a] and Wai-Yeung Wong*[b]*

[a] Institute of Information Photonics Technology, School of Physics and Optoelectronic Engineering, Beijing University of Technology, Beijing 100124, P. R. China

[b] Department of Applied Biology and Chemical Technology and Research Institute for Smart Energy, The Hong Kong Polytechnic University, Hung Hom, Hong Kong, P. R. China; The Hong Kong Polytechnic University Shenzhen Research Institute, Shenzhen 518057, P. R. China

[c] Key Lab of Organic Optoelectronics and Molecular Engineering of Ministry of Education, Department of Chemistry, Tsinghua University, Beijing 100084, P. R. China

Email: duanl@mail.tsinghua.edu.cn (Lian Duan); wai-yeung.wong@polyu.edu.hk (Wai-Yeung Wong)

Table of Contents

SI-1. Synthesis and Characterization

SI-2. Theoretical Calculations

SI-3. Nuclear Magnetic Resonance Spectra

SI-4. Measurements

SI-5. Photophysical Properties

SI-6. Device Fabrication

SI-1. Synthesis and Characterization

All the reagents and solvents used for the synthesis were purchased from commercial suppliers and used without further purification.

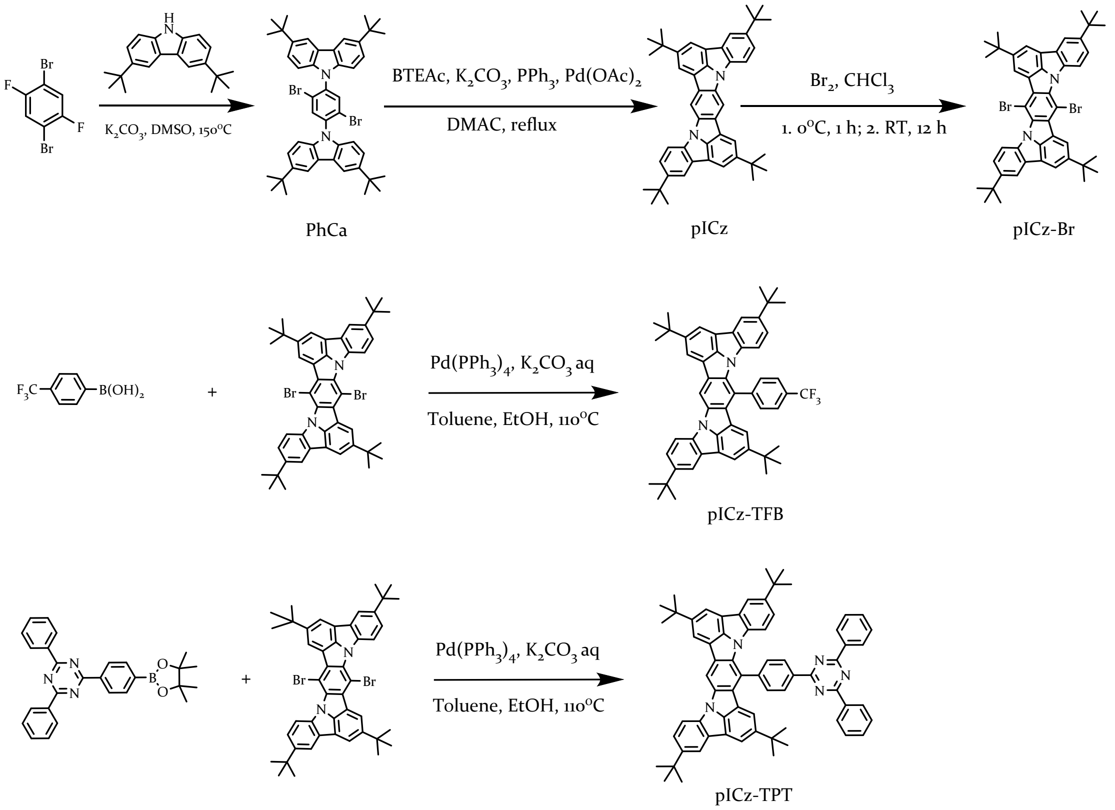

Scheme S1. The synthetic routes for pICz-TFB and pICz-TPT.

Synthesis of PhCa

In a N_2 atmosphere, 3,6-di-*tert*-butyl-carbazole (0.61 g, 2.2 mmol), dibromo-difluoro-benzene (0.27 g, 1 mmol), and K_2CO_3 (0.37 g, 2.7 mmol) were mixed in dimethyl sulfoxide (DMSO, 5 mL). The system was heated to 150 °C and then stirred for 12 h. After cooling to room temperature, the mixture was poured into deionized water. The precipitate was filtered and washed with water.[1] The crude product was purified by silica gel chromatography (hexane: dichloromethane = 3: 1) to afford the product as a white solid (0.59 g, 76%). ^{1}H NMR (400 MHz, Chloroform-*d*) δ (ppm): 8.18 (d, J = 2.0 Hz, 4H), 7.94 (s, 2H), 7.54 (dd, J = 8.6, 1.9 Hz, 4H), 7.18 (d, J = 8.6 Hz, 4H), 1.50 (s, 36H).

Synthesis of pICz

A mixture of 9,9'-(2,5-dibromo-1,4-phenylene)bis(3,6-di-*tert*-butyl-9*H*-carbazole) (0.79 g, 1 mmol), benzyl triethylammonium chloride (0.46 g, 2 mmol), K_2CO_3 (1.34 g, 10 mmol), triphenylphosphine (0.26 g, 2.8 mmol) and palladium(II) acetate (0.67 g, 3 mmol) in 15 mL *N,N*-dimethylacetamide (DMAC) was stirred and heated to reflux for

10 h under a N_2 atmosphere. Then it was cooled to room temperature and poured into ice-water. The resulting precipitate was filtered to give the crude product as a dark green solid and then washed with deionized water and EtOH.[2] The residue was purified by silica gel chromatography (hexane: dichloromethane = 1:1) to afford the product as a yellow solid (0.46 g, 73%). ^{1}H NMR (400 MHz, Chloroform-*d*) δ (ppm): 8.54 (s, 2H), 8.28 (s, 2H), 8.20 (s, 4H), 7.97 (d, J = 8.5 Hz, 2H), 7.64 (dd, J = 8.4, 1.9 Hz, 2H), 1.64 (s, 18H), 1.52 (s, 18H).

Synthesis of pICz-Br

A solution of Br_2 (0.27 mL, 5 mmol) in 10 mL $CHCl_3$ was added dropwise to a suspension of pICz (1.26 g, 2 mmol) in 100 mL $CHCl_3$ at 0 °C. The resulting mixture was stirred for 1 h at 0 °C and stirred at room temperature for another 12 h. After the reaction was complete, the reaction mixture was treated with saturated sodium sulfite solution. The organic layer was collected and washed with excess deionized water. The organic solvent was evaporated and recrystallized using $CHCl_3$/EtOH to give the crude product as a yellow solid.[2] The residue was purified by silica gel chromatography (hexane: dichloromethane = 1:1) to afford the product as a yellow solid (1.29 g, 82.2%).^{1}H NMR (400 MHz, Chloroform-*d*) δ (ppm): 8.94 (d, J = 8.9 Hz, 2H), 8.72 (s, 2H), 8.17 (s, 2H), 8.11 (d, J = 2.1 Hz, 2H), 7.56 (dd, J = 9.0, 2.1 Hz, 2H), 1.63 (s, 19H), 1.52 (s, 18H).

SI-2. Theoretical Calculations

The density functional theory (DFT) and time-dependent DFT (TD-DFT) calculations were conducted using Gaussian program with b3lyp/6-31g(d) basis set.

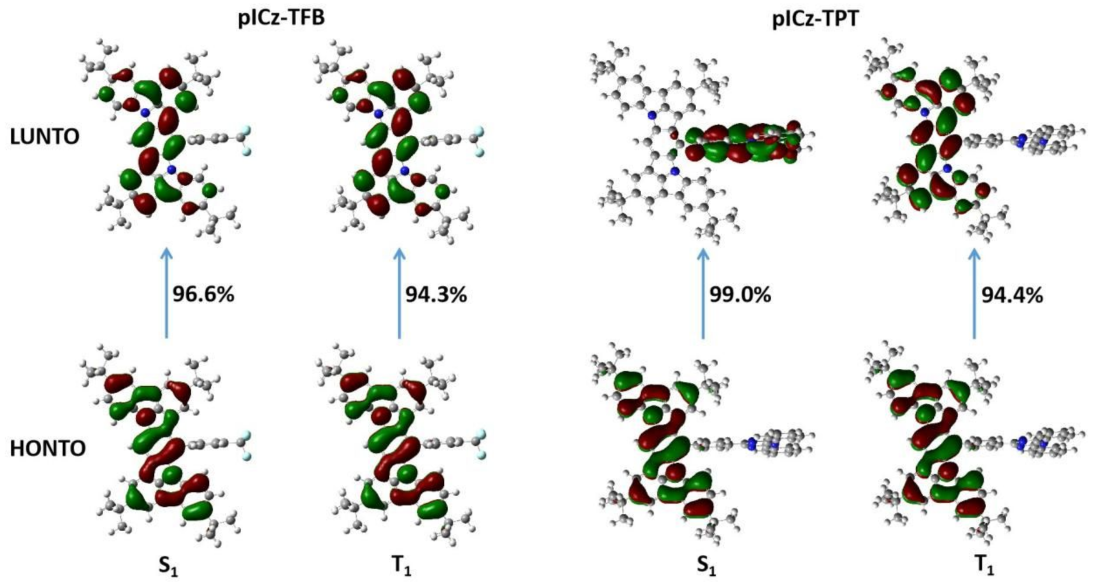

Figure S1. NTO distributions for the $S_0 \rightarrow S_1$ and $S_0 \rightarrow T_1$ transitions of pICz-TFB and pICz-TPT.

SI-3. Nuclear Magnetic Resonance Spectra

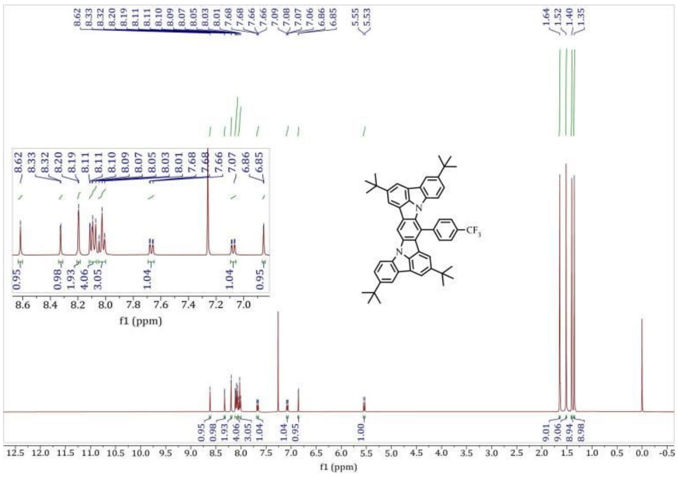

Figure S2. ^{1}H NMR spectrum of pICz-TFB in $CDCl_3$.

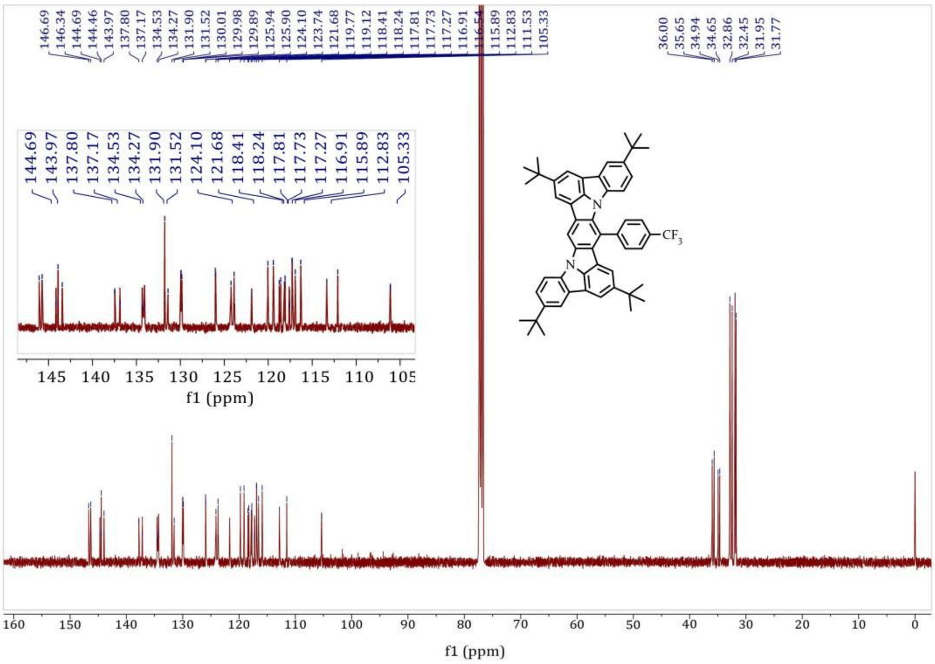

Figure S3. $^{13}C\{^{1}H\}$ NMR spectrum of pICz-TFB in $CDCl_3$.

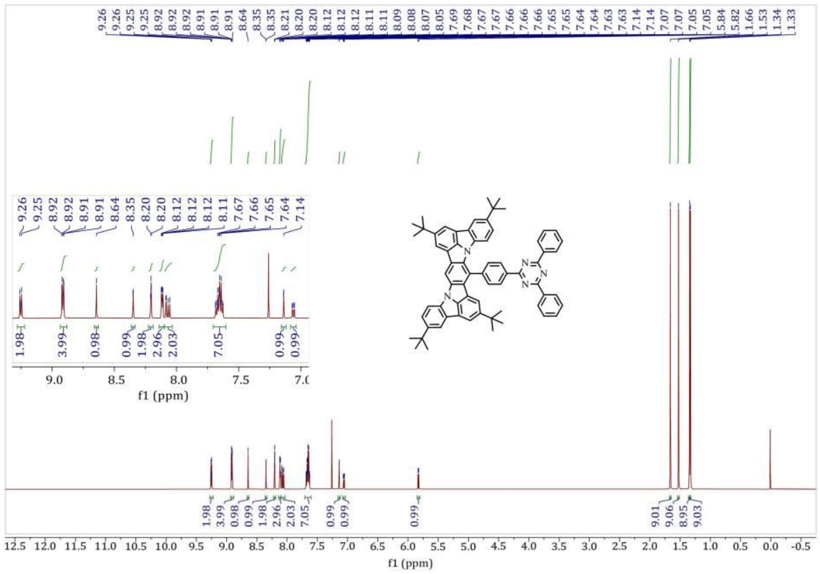

Figure S4. ^{1}H NMR spectrum of pICz-TPT in $CDCl_3$.

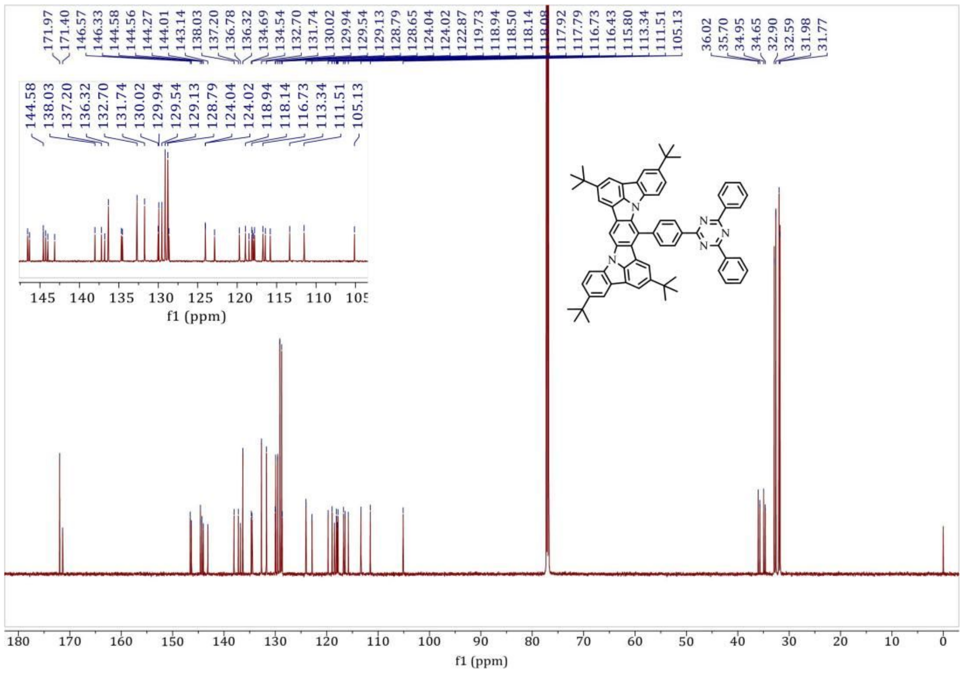

Figure S5. $^{13}C\{^{1}H\}$ NMR spectrum of pICz-TPT in $CDCl_3$.

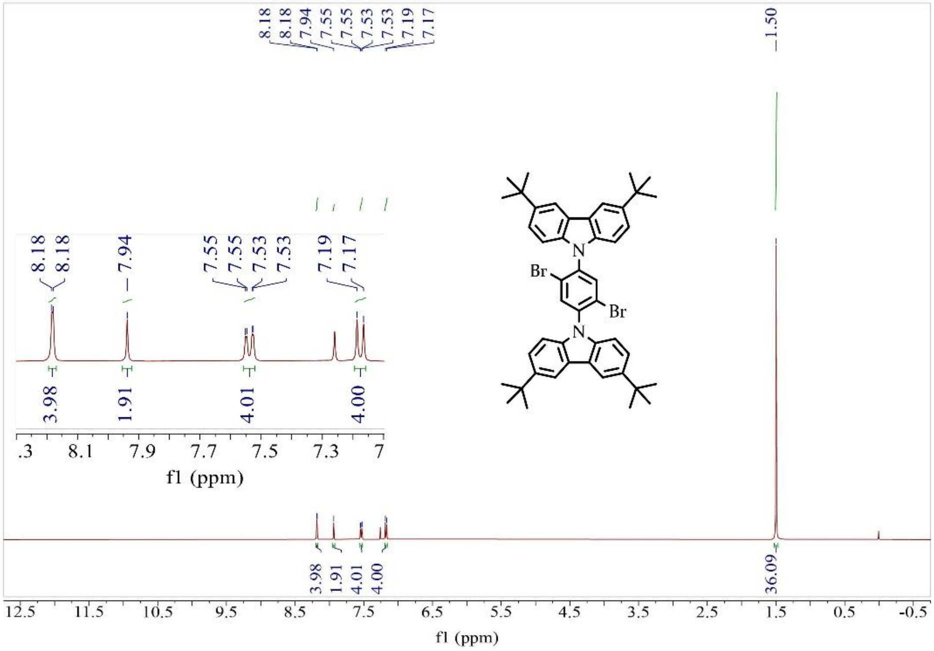

Figure S6. ^{1}H NMR spectrum of PhCa in $CDCl_3$.

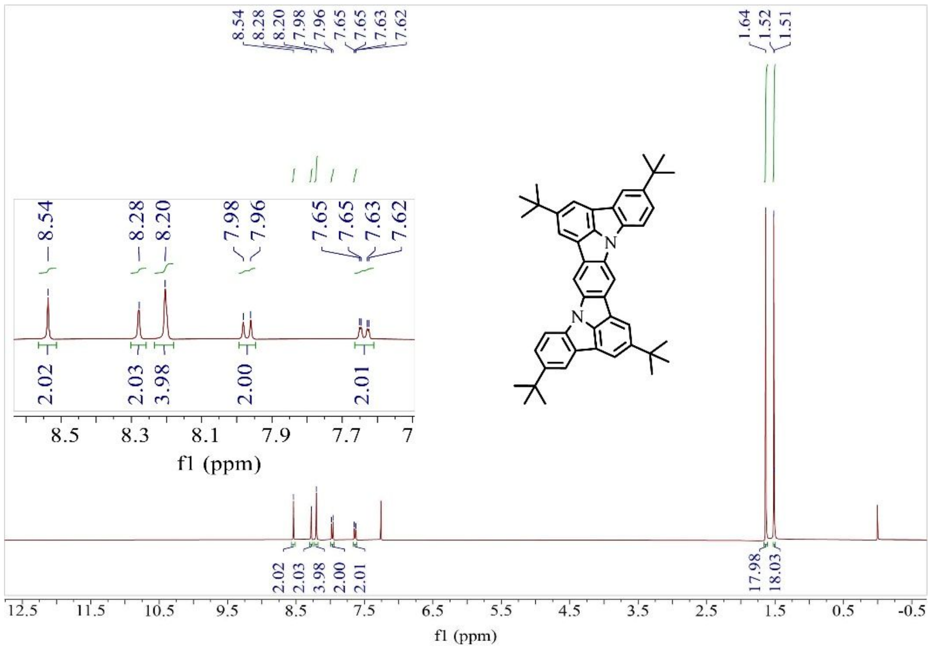

Figure S7. ^{1}H NMR spectrum of pICz in $CDCl_3$.

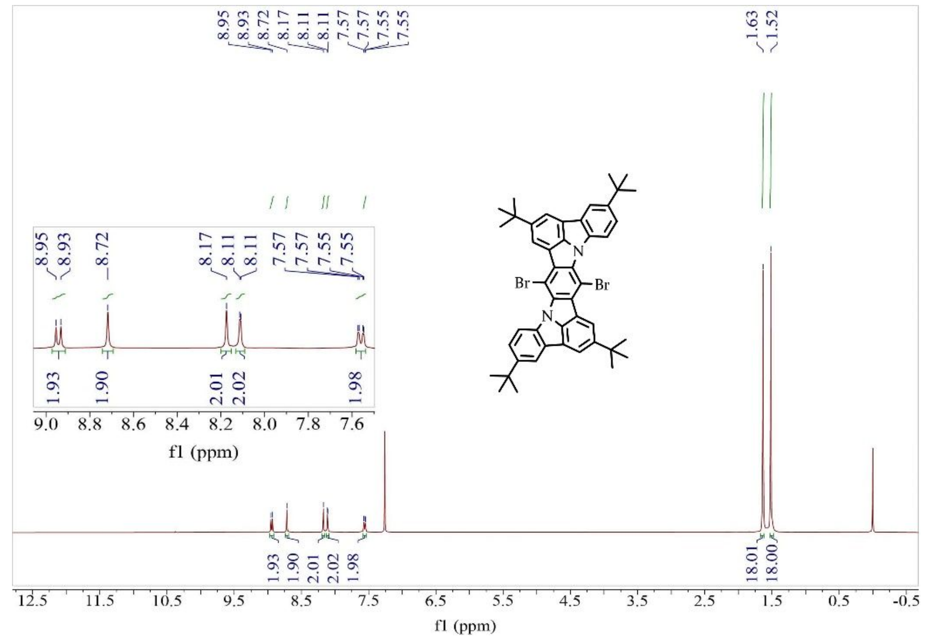

Figure S8. ^{1}H NMR spectrum of pICz-Br in $CDCl_3$.

SI-4. Measurements

Instrumentation for structure characterization: ^{1}H and ^{13}C nuclear magnetic resonance (NMR) spectra were recorded on a Bruker Ultra Shield Plus 400 MHz instrument (400 MHz for ^{1}H and 101 MHz for ^{13}C, respectively) with chloroform-*d* ($CDCl_3$) as the solvent and tetramethylsilane (TMS, $\delta = 0.00$ ppm) as the internal standard. Molecular mass was determined by Bruker Ultraflextreme MALDI-TOF mass spectrometer.

Thermal stability measurements: Thermal properties of the blue emitters were investigated by thermogravimetric analysis (TGA). TGA measurements were performed using a Perkin Elmer Pyris 6 under a nitrogen atmosphere with a heating rate of 10°C min^{-1} from 30 to 800°C.

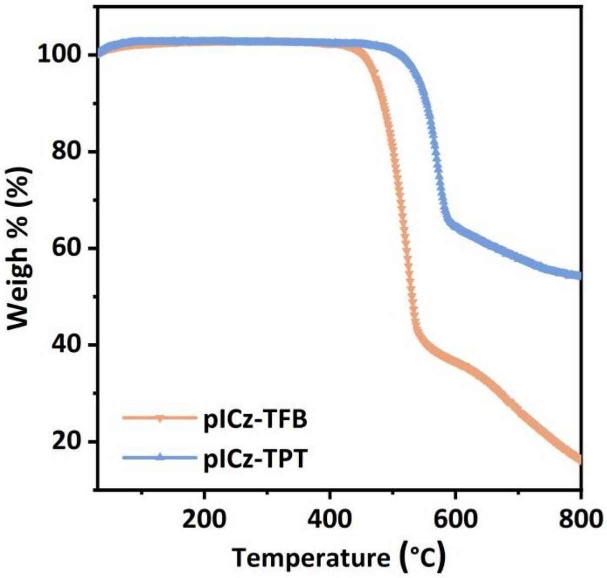

Figure S9. TGA curves of pICz-TFB and pICz-TPT.

Electrochemical property measurements: Cyclic voltammetry (CV) measurements were performed at room temperature on a CHI660E system in a typical three-electrode cell with a working electrode (glass carbon), a reference electrode (Ag/Ag^+, referenced against ferrocene/ferrocenium (FOC), and a counter electrode (Pt wire) in acetonitrile solution of tetrabutylammonium hexafluorophosphate (Bu_4NPF_6) (0.1 M) at a sweeping rate of 100 mV s^{-1}. The highest occupied molecular orbital (HOMO) energy levels (E_{HOMO}) of the materials were estimated based on the reference energy level of ferrocene (4.8 eV below the vacuum) according to Equation S1:

$$\mathrm{E_{HOMO}} = -\left(\mathrm{E^{Ox}_{onset}} - E_{(\mathrm{Fc/Fc^+})} + 4.8\right)\ \mathrm{eV} \quad \text{(S1)}$$

where $E_{(Fc/Fc^+)}$ is the onset potential of oxidation wave of ferrocene (Fc) *vs* Ag/Ag^+ and $\mathrm{E^{Ox}_{onset}}$ is the onset potential of the oxidation wave of the materials deposited as thin films on the surface of the working electrode. The lowest unoccupied molecular orbital (LUMO) energy level (E_{LUMO}) was estimated by adding the optical bandgap (E_{g}) to the corresponding HOMO energy level.

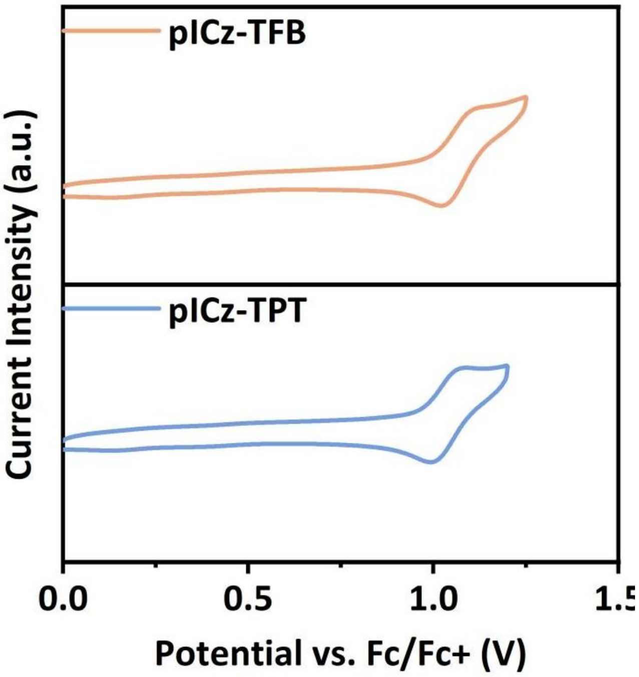

Figure S10. Cyclic voltammograms of pICz-TFB and pICz-TPT.

Photophysical property measurements: Ultraviolet-visible (UV-Vis) spectra were recorded on a Varian Cary 4000 UV-Visible spectrophotometer. The photoluminescent (PL) spectra were obtained on an Fluoromax-4 spectrophotometer. The phosphorescent spectra were measured at 77 K using liquid nitrogen with a 5 ms delay. Transient PL decay curves were collected using an Edinburgh FLS 1000 fluorescence spectrophotometer at temperatures of 100 K, 200 K and 300 K. The absolute photoluminescence quantum yield (PLQY) was obtained on an Edinburgh FLS1000 fluorescence spectrophotometer with an integrating sphere.

SI-5. Photophysical Properties

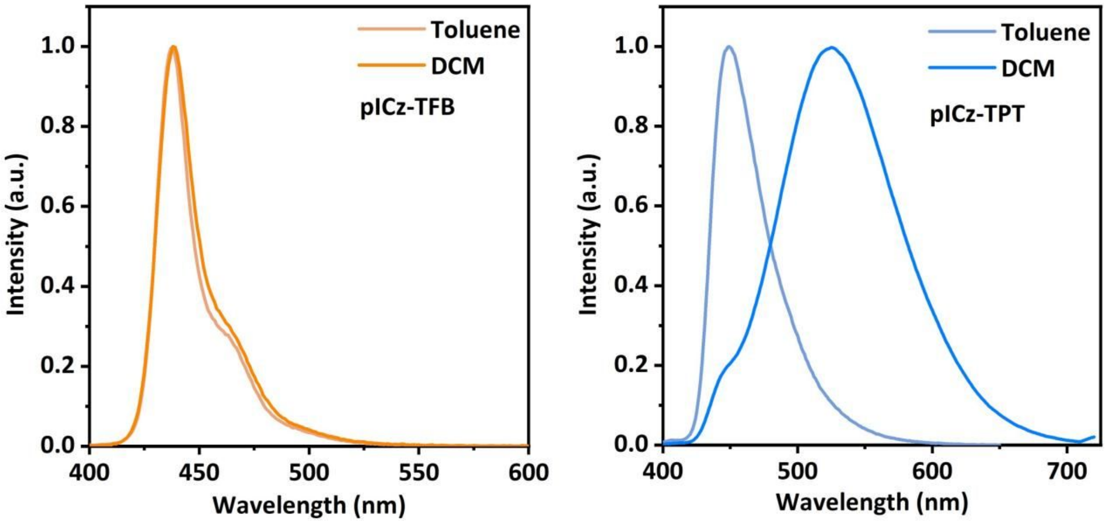

Figure S11. The normalized fluorescence spectra of pICz-TFB and pICz-TPT in

toluene and dichloromethane (DCM) solutions (10^{-5} mol L^{-1}).

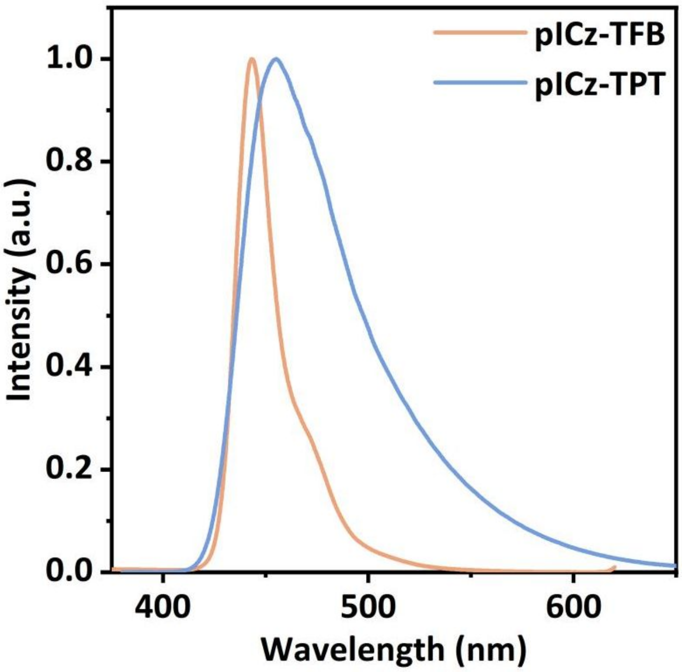

Figure S12. The photoluminescence spectra of doped films (PPF: 1% pICz-TFB and PPF: 1% pICz-TPT).

Table S1. Critical parameters for previously reported pICz derivatives.

Compounds	ΔE_{ST} (eV)^a	k_{RISC} (s^{-1})^b	EQE_{max} (%)^c	Ref
pICz-TFB	0.33	9.7×10^2	2.2	This work
pICz-TPT	0.25	1.1×10^4	14.4	This work
pICz	0.29	-	-	[2]
pICz-TPA	0.32	-	-	[2]
tPBisICz	0.27	1.4×10^3	-	[1]
tBisICz	0.29	1.5×10^2	-	[1]
DiICzMes4	0.26	1.8×10^2	2.0	[3]
pICz-PPO	0.35	-	1.9	[4]
pICz-2PPO	0.36	-	3.2	[4]

^{a} Energy gap between the lowest excited singlet and triplet states;

^{b} Reverse intersystem crossing rate;

^{c} Maximum external quantum efficiency of the non-sensitized OLEDs

Table S2. The kinetic rate constants of pICz-TFB and pICz-TPT.

	k_r (s^{-1})	k_{nr} (s^{-1})	k_{ISC} (s^{-1})	k_{RISC} (s^{-1})
pICz-TFB	5.36×10^7	5.36×10^7	4.17×10^7	9.71×10^2
pICz-TPT	5.15×10^7	3.78×10^7	1.56×10^8	1.11×10^4

Calculation of the kinetic rate constants[5]:

$$k_r = \phi_{PF} / \tau_p \quad \text{(S2)}$$

$$\phi = k_r / (k_r + k_{nr}) \quad \text{(S3)}$$

$$\phi_{PF} = k_r / (k_r + k_{ISC} + k_{nr}) \quad \text{(S4)}$$

$$\phi_{ISC} = 1 - \phi_{PF} \quad \text{(S5)}$$

$$k_{RISC} = k_p k_d \phi_{TADF} / k_{ISC} \phi_{PF} \quad \text{(S6)}$$

$$k_P = 1/\tau_p \quad \text{(S7)}$$

$$k_d = 1/\tau_d \quad \text{(S8)}$$

where k_r, k_{nr}, k_{ISC} and k_{RISC} represent the rate constant of radiative, non-radiative, intersystem crossing and reverse intersystem crossing, respectively. ϕ, ϕ_{PF}, ϕ_{TADF}, τ_p, and τ_d, are obtained directly from the PLQY measurement and the exponential fitting parameters of the transient photoluminescence decay spectra, representing total PLQY, quantum yield of the prompt component, quantum yield of the delayed component, lifetimes of the prompt and delayed components, respectively.

SI-6. Device Fabrication

Before device fabrication, the ITO glass substrates were pre-cleaned carefully. Then the sample was transferred to the deposition system. The devices were prepared in vacuum at a pressure of 5×10^{-6} Torr. The organic layers were thermally evaporated at a rate of 1.0 Å s^{-1}. After the organic film deposition, 0.5 nm of LiF and 150 nm of aluminum were thermally evaporated onto the organic surface. All the organic materials used were purified by a vacuum sublimation approach. The electrical characteristics of the devices were measured with a Keithley 2400 source meter. The electroluminescence spectra and luminance of the devices were obtained on a PR650 spectrometer. All the device fabrication and characterization steps were carried out at room temperature under ambient laboratory conditions.

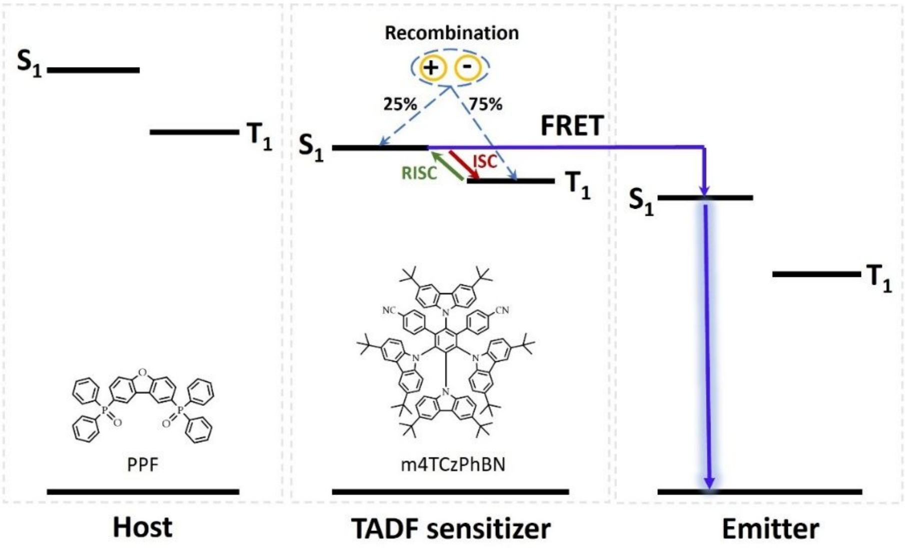

Figure S13. The scheme of energy transfer process of PPF:30% m4TCzPhBN:1% pICz-TFB / pICz-TPT emitting layer in the hyperfluorescent OLEDs.

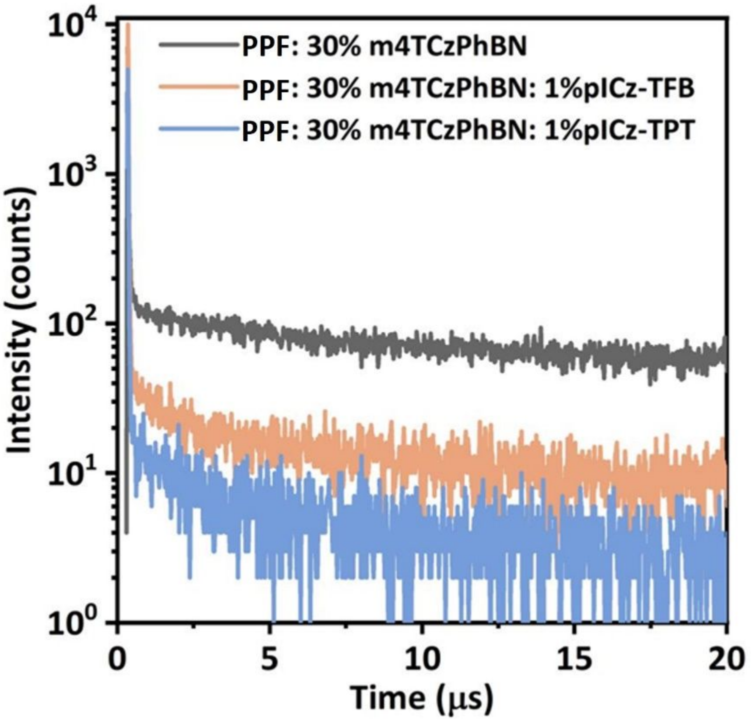

Figure S14. The photoluminescence decay curves of PPF:30% m4TCzPhBN, PPF:30% m4TCzPhBN:1% pICz-TFB and PPF:30% m4TCzPhBN:1%pICz-TPT.

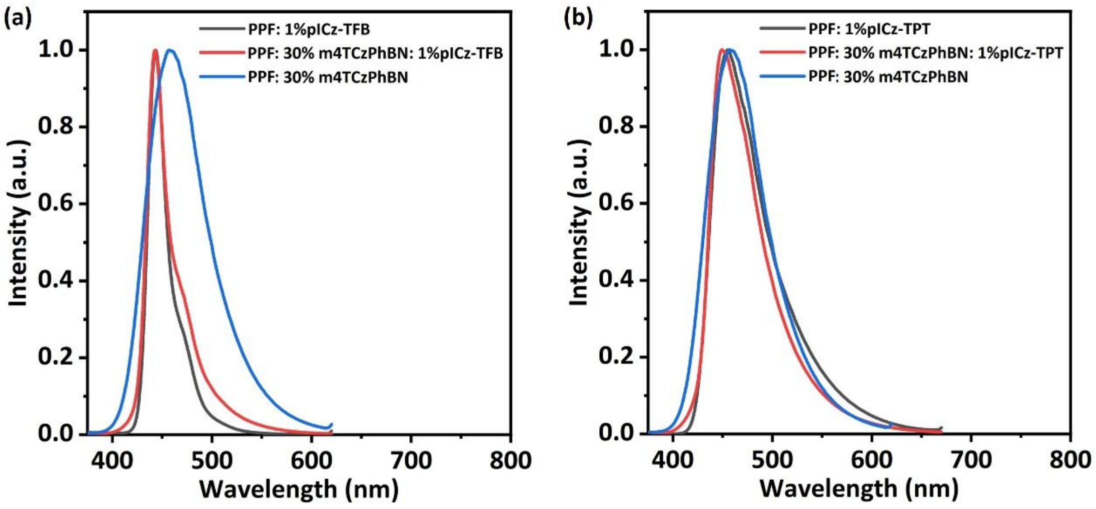

Figure S15. The photoluminescence spectra of the doped films of (a) PPF:30% m4TCzPhBN, PPF:1% pICz-TFB, PPF:30% m4TCzPhBN:1% pICz-TFB, and (b) PPF:30% m4TCzPhBN, PPF:1% pICz-TPT, PPF:30% m4TCzPhBN:1% pICz-TPT.

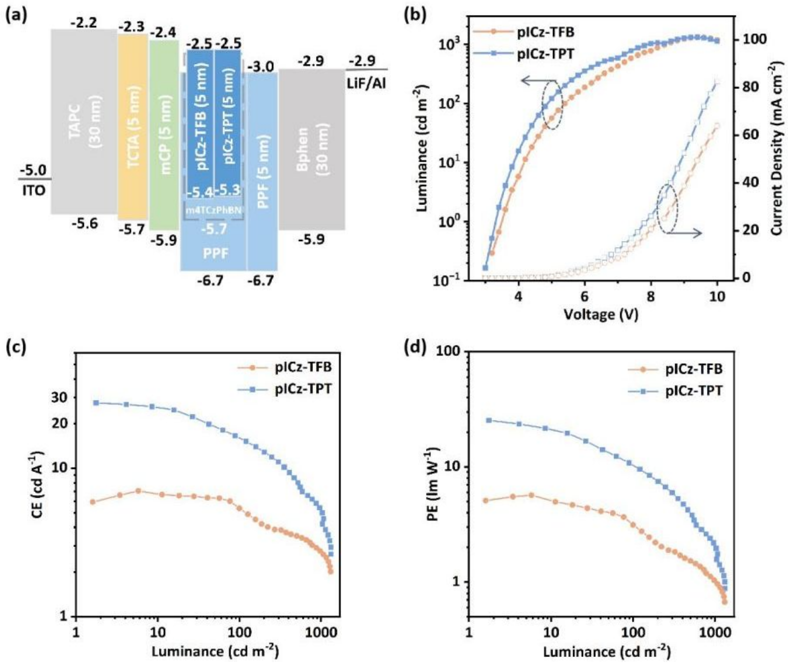

Figure S16. (a) Energy level diagram, (b) luminance-voltage-current density plots, (c) current efficiency-luminance plots and (d) power efficiency-luminance plots of the hyperfluorescent OLEDs.

Table S3. External quantum efficiency and efficiency roll-off of OLEDs and HF OLEDs based on pICz-TFB and pICz-TPT.

Device	Emitter	EQE (%)			Roll-off (%)	
		Max	100 cd m^{-2}	1000 cd m^{-2}	100 cd m^{-2}	1000 cd m^{-2}
OLEDs	pICz-TFB	2.2	-	-	-	-
	pICz-TPT	14.4	2.9	-	80	-
HF OLEDs	pICz-TFB	12.0	6.4	2.5	47	79
	pICz-TPT	24.2	12.3	4.3	49	82
	pICz[2]	32.0	6.8	3.5	68	89

References

(1) Patil, V. V.; Lee, H. L.; Kim, I.; Lee, K. H.; Chung, W. J.; Kim, J.; Park, S.; Choi, H.; Son, W.; Jeon, S. O.; Lee, J. Y. Purely Spin-Vibronic Coupling Assisted Triplet to Singlet Up-Conversion for Real Deep Blue Organic Light-Emitting Diodes with Over 20% Efficiency and y Color Coordinate of 0.05. *Adv. Sci.* **2021**, *8*, 2101137.

(2) Wei, J. B.; Zhang, C.; Zhang, D. D.; Zhang, Y. W.; Liu, Z. Y.; Li, Z. Q.; Yu, G.; Duan, L. Indolo[3,2,1-*Jk*]Carbazole Embedded Multiple-Resonance Fluorophors for Narrowband Deep-blue Electroluminescence with EQE≈34.7% and CIE_y≈0.085. *Angew. Chem. Int. Ed.* **2021**, *60*, 12269-12273.

(3) Hall, D.; Stavrou, K.; Duda, E.; Danos, A.; Bagnich, S.; Warriner, S.; Slawin, A. M. Z.; Beljonne, D.; Köhler, A.; Monkman, A.; Olivier, Y.; Zysman-Colman, E. Diindolocarbazole – Achieving Multiresonant Thermally Activated Delayed Fluorescence without the Need for Acceptor Units. *Mater. Horiz.* **2022**, *9*, 1068-1080.

(4) Wang, S. X.; Zhou, J. P.; Jin, J. B.; Mai, M. Q.; Tsang, C.-S.; Lee, L. Y. S.; Duan, L.; Wong, W.-Y. Acceptor Modification of Diindolocarbazole Embedded Multiple-Resonance Emitters for Efficient Narrowband Deep-Blue OLEDs with $CIE_y \leq 0.08$ and Alleviated Efficiency Roll-Off. *J. Mater. Chem. C* **2024**, *12*, 2485-2492.

(5) Gan, Y.; Peng, X.; Qiu, W.; Wang, L.; Li, D.; Xie, W.; Liu, D.; Li, M.; Lin, J.; Su, S. J. Multiple charge transfer disk-like emitters with fast fluorescence radiation rate and high horizontal dipole orientation for pure blue organic light-emitting diodes. *Chem. Eng. J.* **2022**, *430*, 133030.
